# Successful management of severe asthma in a young boy with eosinophilic chronic rhinosinusitis who received omalizumab: a case report

**DOI:** 10.1186/s13223-019-0369-7

**Published:** 2019-09-05

**Authors:** Yasuho Shoda, Misa Watanabe, Kota Wada, Takehiko Soutome, Yumiko Komine, Tetsuo Mikami, Tetsuo Nemoto, Akira Ohara

**Affiliations:** 1 Department of Pediatrics, Toho University School of Medicine, 6-11-1, Omori-Nishi, Ota-ku, Tokyo, 143-8541 Japan; 2 Department of Otolaryngology, Toho University School of Medicine, 6-11-1, Omori-Nishi, Ota-ku, Tokyo, 143-8541 Japan; 3 Department of Pathology, Toho University School of Medicine, 6-11-1, Omori-Nishi, Ota-ku, Tokyo, 143-8541 Japan; 4 Department of Pathology, Showa University School of Medicine, Yokohama Northern Hospital, 35-1 Chigasaki-Chuo, Tsuzuki-ku, Yokohama, Kanagawa, 224-8503 Japan

**Keywords:** Bronchial asthma, Child, Eosinophilic chronic rhinosinusitis, Nasal polyp, Omalizumab, Mepolizumab

## Abstract

**Background:**

The incidence of chronic rhinosinusitis with nasal polyps has recently increased in Japan and other East Asian countries, and this disease is called eosinophilic chronic sinusitis (ECRS) in Japan. ECRS usually occurs in adults and is frequently accompanied by refractory bronchial asthma. However, its occurrence in children under 10 years of age is rare. Here, we present an unusual case of ECRS complicated by intractable asthma in an 8-year-old boy.

**Case presentation:**

Oral administration of prednisolone (10 mg/day) initially relieved the ECRS and bronchial asthma, but both returned during prednisolone dose reduction. Because nasal cavity-opening surgery was ineffective, oral administration prednisolone at 10 mg/day was continued. Pancytopenia was observed 16 months after the start of treatment, and the patient was admitted to our hospital. He was diagnosed with refractory cytopenia in childhood, but gradually improved after cyclosporine treatment. Although the dose of cyclosporine was therapeutic for asthma, it did not alleviate the asthma attacks, and the patient’s quality of life markedly decreased. We administered omalizumab even though its use was contraindicated by negative results in an inhalable antigen test. After the third administration of omalizumab, the asthma was better controlled and respiratory function improved; however, the nasal symptoms of ECRS persisted. Attempts to relieve these symptoms by increasing the therapeutic dose of omalizumab were only partially successful. We replaced omalizumab with mepolizumab; doing so slightly improved the sinusitis symptoms, but quality of life remained unsatisfactory. We repeated the nasal cavity-opening surgery. After surgery, the asthma and sinusitis were unchanged.

**Conclusions:**

Omalizumab effectively treated the severe combined asthma in a young patient, but its effect on sinusitis was insufficient. More cases and long-term follow-up data are needed to better evaluate the effectiveness of mepolizumab for treatment of ECRS.

## Background

Eosinophilic sinusitis, also known as eosinophilic chronic rhinosinusitis (ECRS) in Japan, is an inflammatory condition involving the nose and paranasal sinuses. It is characterized by the presence of multiple nasal polyps, olfactory dysfunction, and intractable sinusitis with eosinophilia in the nasal mucosa and peripheral blood. Macrolide antibiotics, which are generally used for the treatment of sinusitis, are ineffective, and oral steroids are generally required. Recurrence is common, even in cases treated by endoscopic sinus surgery. This condition is often complicated by refractory asthma, particularly aspirin-induced asthma [1]. The incidence of chronic sinusitis with nasal polyps has recently increased in Japan and other regions in East Asia [2]. Although ECRS usually occurs in adult men, there are a few reported cases in patients under 15 years old [1]. We present a case of ECRS in an 8-year-old boy, in which refractory asthma was successfully treated with omalizumab and reduced doses of systemic steroids.

## Case presentation

Written informed consent was obtained from the patient’s parents for publication of this case report and any accompanying images.

An 8-year-old boy presented to our hospital in October 2013 with continuous wheezing for 1 month. His asthma had progressively increased in severity beginning 10 months earlier, when he had had pneumonia, which recurred twice. He was treated with montelukast sodium, fluticasone propionate/salmeterol xinafoate (100 μg q.i.d.), epinastine hydrochloride, and mometasone furoate hydrate (b.i.d.) by his pediatrician, with no effect.

The patient’s medical history included recurrent acute otitis media and seasonal allergic rhinitis (AR), both beginning when he was 5 years-old. He was diagnosed with bronchial asthma (BA) when he was 6 years old, when AR became continuous and severe. He lived with his parents and younger brother, all of whom exhibited BA and pollinosis. There were no pets or smokers in his house.

On physical examination, the patient appeared to be well. His weight was 21.9 kg (− 1.0 SD) and his height was 126.6 cm (− 0.2 SD). His vital signs were as follows: pulse rate, 81 beats per minute; respiratory rate, 20 breaths per minute; temperature, 37.0 °C; and oxygen saturation, 99% while breathing ambient air. Examination of the chest revealed prolonged expirations, sibilant rhonchi, and coarse crackles on the right side. Spirometry showed a forced vital capacity of 92%, a 1-s forced expiratory volume of 64%, and poor reversibility upon inhalation of a β stimulant.

Hematological and serum biochemical examinations revealed a white blood cell count of 8600/μL, with a differential profile of 23% eosinophils, and a serum total IgE level of 151 IU/mL. ImmunoCAP tests for IgEs produced in response to *Dermatophagoides pteronyssinus*, *Aspergillus*, *Candida*, *Alternaria*, *Cladosporium*, and other common allergens were all negative.

## Outcome and follow-up

**Fig. 1:**
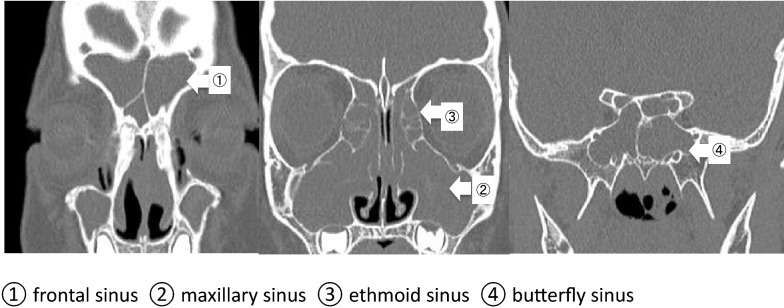
Paranasal computed tomography images before treatment. Pretreatment computed tomography at 8 years and 6 months of age shows paranasal sinusitis. The scans reveal secretory reservoirs in the frontal, maxillary, ethmoid, and butterfly paranasal sinuses

**Fig. 2:**
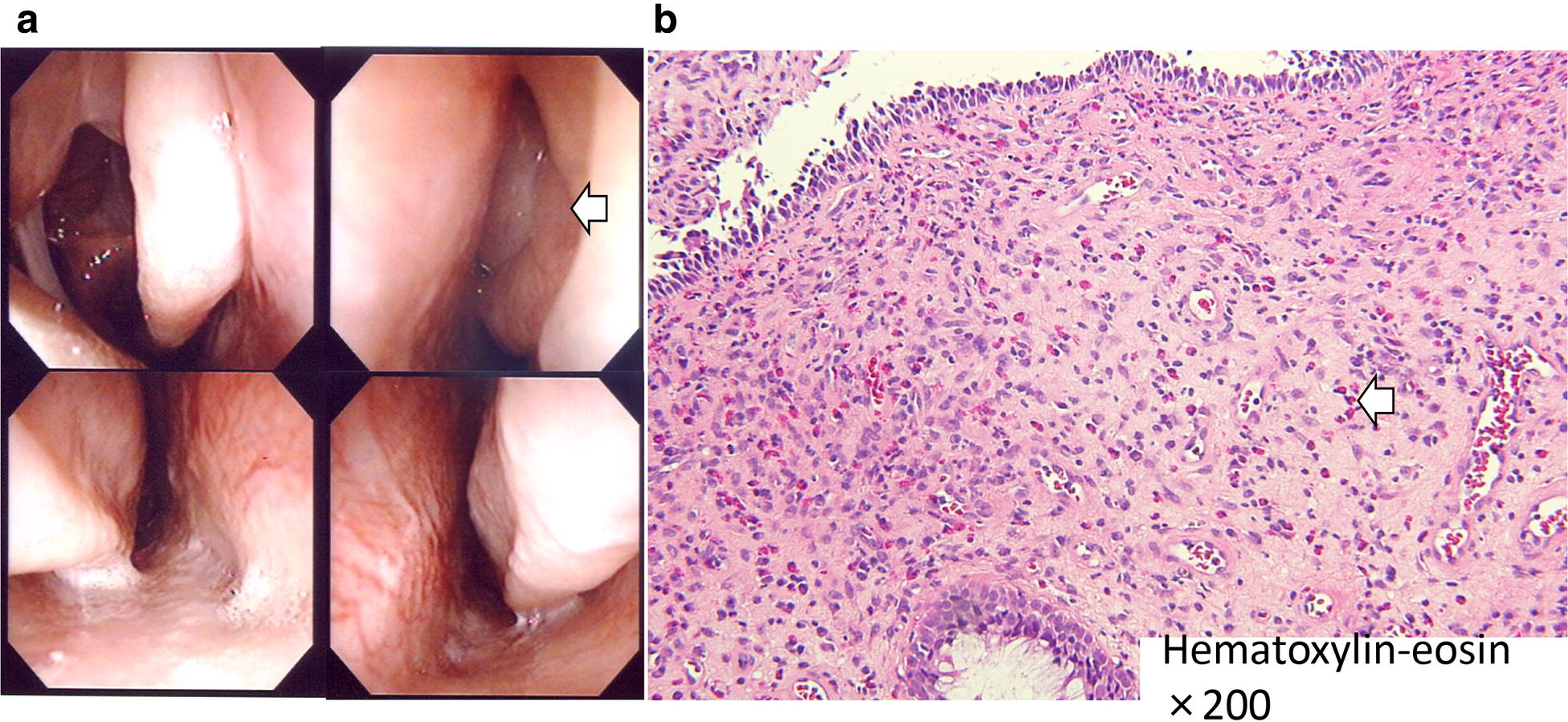
Endoscopic images of the bilateral nasal cavity and histological analysis of the nasal polyps. **a** The endoscopic images show polyps in the bilateral nasal cavity. **b** Histological analysis shows numerous eosinophils in the polyps. Hematoxylin and eosin staining ×200. All paranasal sinuses were released, but after 4 months later, the nasal polyps recurred

Eosinophilia, bilateral nasal polyps, and pansinusitis were observed via computed tomography (Fig. 1), and the diagnosis was severe refractory BA associated with ECRS. The patient received oral prednisolone (PSL, 10 mg/day), which alleviated both the BA and nasal symptoms. However, during tapering of the steroid, the symptoms soon recurred, and nasal-opening surgery (endoscopic sinus surgery) was performed half a year later. After surgery, the BA was controlled during PSL dose reduction, but 4 months after surgery, the nasal polyps recurred and an eosinophilic infiltrate was found in the extracted nasal polyps (Fig. 2). Therefore, PSL at 10 mg/day was continued.

Pancytopenia was observed in the spring of 2015, at which time the boy was 10 years-old, and progressed relatively rapidly. A bone marrow examination showed hypoplastic marrow with mild dysplasia, and refractory cytopenia childhood (World Health Organization 2008 classification) was diagnosed. There was no report of pancytopenia associated with ECRS. To prevent drug-induced pancytopenia, all drugs except fluticasone propionate/salmeterol xinafoate and oral PSL were discontinued; however, the bone marrow failure was not resolved. After the pancytopenia had progressed, anemia symptoms were observed in addition to the original loss of respiratory function, and quality of life (QOL) markedly decreased.

**Fig. 3:**
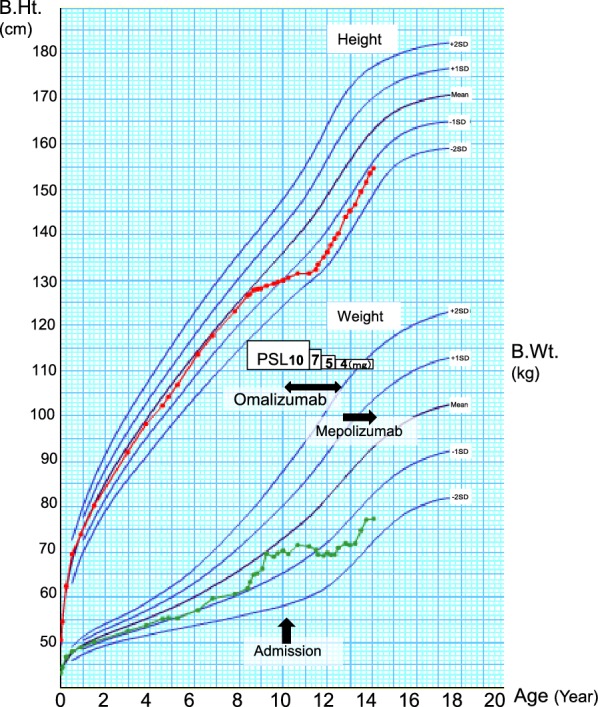
Growth curve of the patient height and weight. The effects of oral steroids suppressed the patient height growth and gained weight. His growth has been restored as the symptoms improved with the administration of omalizumab and he was able to lose weight on oral steroids. B.Ht. and B.Wt. indicate body height, and body weight, respectively

Since cytopenia caused by the immunopathology was indicated by the patient’s history of ECRS, oral cyclosporine (CsA) (5 mg/kg/day) was administered. Blood cell numbers tended to increase beginning 2 months thereafter. After 7 months, the thrombocytopenia and anemia improved; this recovery trend was confirmed by bone marrow examination. However, the BA-associated symptoms did not improve, even after administering CsA at a therapeutic dose for BA. Although contraindicated for omalizumab owing to lack of in vitro reactivity to a perennial aeroallergen, the patient received this agent after his parents provided informed consent. Three months after initiating omalizumab treatment, both his symptoms and respiratory function definitely improved. As we were concerned that long-term oral steroid administration might slow his growth, we started tapering PSL and CsA after 9 months (Fig. 3).

**Fig. 4:**
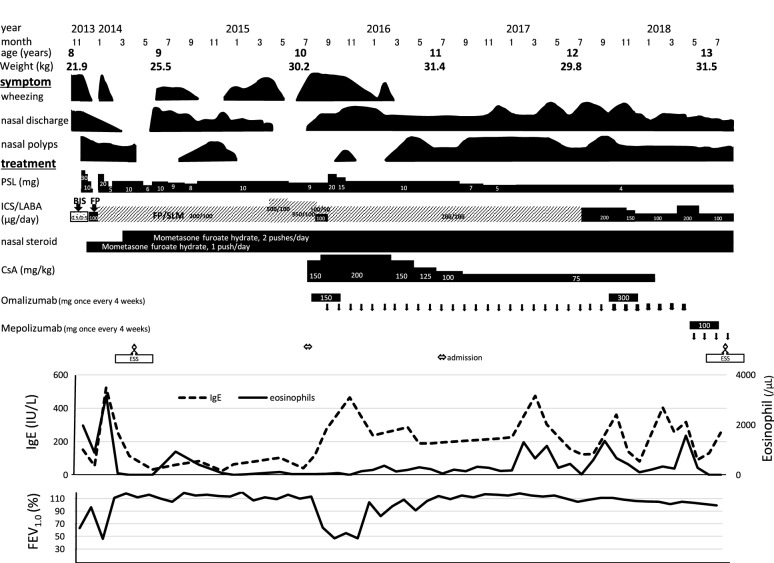
Clinical course and laboratory findings. A timeline of the treatments, symptoms, IgE levels, eosinophil counts, and %FEV_1.0_ is presented. *PSL* prednisolone, *ICS/LABA* inhaled long-acting β-agonist/corticosteroid, *CsA* cyclosporine, *BIS* budesonide inhalation suspension, *FP* fluticasone propionate, *SLM* salmeterol xinafoate, *ESS* endoscopic sinus surgery

The patient is currently 13 years-old, and no longer requires CsA. The dose of PSL now can be reduced to 4 mg/day, and his height is catching up. Omalizumab therapy was continued until he was 12 years and 10 months of age; the asthma symptoms were well controlled, and cytopenia was not observed. However, because he still suffered from the nasal symptoms caused by ECRS, we increased the therapeutic dose of omalizumab from 150 to 300 mg once every 4 weeks, which was only slightly effective. One month before his thirteenth birthday, we substituted mepolizumab for omalizumab, which slightly improved the symptoms of sinusitis but not QOL. We again performed nasal-opening surgery. After surgery, the asthma and sinusitis remain. A flow chart showing the treatments and responses is presented in Fig. 4.

## Discussion and conclusions

Eosinophilic sinusitis was defined by Haruna in Japan in 2001 [3]. Guidelines were formulated by Tokunaga et al. [4] and diagnostic criteria were established [4]. We presented a case of pediatric ECRS that meets the required criteria and represents the most severe type of ECRS in young children. In addition, we successfully limited the systemic administration of steroids by treating our patient with omalizumab.

Although we could not find any studies of eosinophilic sinusitis in children, there are several reports of sinusitis combined with aspirin-induced asthma in children [5]. Once ECRS becomes widely recognized worldwide, finding similar pathological conditions among children diagnosed with refractory asthma is possible.

Our case is distinguished by its course of onset. Ordinary IgE-related allergic asthma generally results from food allergies and/or atopic dermatitis in early childhood and is generally followed, in order, by BA and AR (“allergy march”) [6]. Our patient’s asthma was diagnosed at the age of 6 years, which is later than the mean onset age of pediatric BA (2.5 years; peak onset, 1–2 years). Prior to the onset of asthma, he had intermittent AR symptoms, which gradually became continuous, as well as three bouts of pneumonia. Both the AR and BA became severe in the autumn of his 8th year. Hence, in terms of the course of events, his asthma differed from ordinary pediatric asthma.

Although the predicted probability of rhinosinusitis recurrence is high [4], we treated the paranasal sinuses via surgery so that the nasal steroid spray treatments could be continued. As a result, we were able to rapidly reduce the oral steroid dose postoperatively. However, 4 months after surgery, we had to increase the dose because of exacerbation of the nasal polyps.

Since our patient was a young child, and to limit the use of steroids (and thus prevent growth disorders and other side effects), we tried treating him with omalizumab. At that time, there were three studies showing that omalizumab was an effective treatment for adult ECRS [7–9]. All reported less severe asthma attacks and nasal symptoms after the fourth administration of this anti-IgE antibody. In our case, the nasal and asthmatic symptoms improved during the early stage of omalizumab administration. However, after reducing the dose of the coadministered steroid, the nasal polyps recurred. Unfortunately, omalizumab effectively treated the asthma, but not the ECRS in our case. We increased the dose of omalizumab as much as possible to compensate for increases in body weight, which modestly improved the nasal symptoms. These findings show that omalizumab needs to be administered in sufficient amounts in order to ameliorate the symptoms of ECRS.

A recent report shows that mepolizumab, an interleukin-5 monoclonal antibody, is an effective treatment for ECRS [10–13]. Because it is not approved for use in children younger than 12 years, we began administering it in place of omalizumab when our patient was 12 years and 11 months of age. Mepolizumab slightly improved his sinusitis symptoms but his QOL was unsatisfactory. More cases and long-term follow-up data are needed to accurately evaluate the effectiveness of mepolizumab.

Our case was complicated by pancytopenia, although we could not find any reports in which pancytopenia coexisted with eosinophilic sinusitis. Eosinophilic sinusitis is thought to be an eosinophilia associated with the respiratory tract. Although its transition to eosinophilic polyangiitis granuloma was possible given the patient’s history of pneumonia and eosinophilic pneumonia, there was no indication that this had occurred.

The therapeutic dose of CsA is the same for pancytopenia and asthma. In our case, however, this dose was effective for pancytopenia but not BA or ECSR. Therefore, we believe that these diseases are caused by different mechanisms and that their co-occurrence is accidental.

It is important for us to keep ECRS in mind, even in young children, when the onset of severe BA atypically precedes the onset of severe chronic rhinosinusitis. Unfortunately, in our case, omalizumab and mepolizumab did not effectively treat the sinusitis, despite improving the asthma symptoms. Being able to decrease the dose of PSL, as done in our study, is essential for allowing the growth rate to return to normal.

In the future, we hope the expand the indications for biologics such as omalizumab and mepolizumab to ECRS and more young children, as they have few side effects and can be effective.

## Data Availability

Data sharing is not applicable to this article as no datasets were generated or analyzed during the current study.

## Abbreviations

AR: allergic rhinitis
BA: bronchial asthma
CsA: cyclosporine
ECRS: eosinophilic chronic rhinosinusitis
PSL: prednisolone
QOL: quality of life
